# Skim resequencing finely maps the downy mildew resistance loci *RPF2* and *RPF3* in spinach cultivars whale and Lazio

**DOI:** 10.1093/hr/uhad076

**Published:** 2023-04-19

**Authors:** Gehendra Bhattarai, Ainong Shi, Beiquan Mou, James C Correll

**Affiliations:** Department of Horticulture, University of Arkansas, Fayetteville, AR 72701, USA; Department of Horticulture, University of Arkansas, Fayetteville, AR 72701, USA; USDA-ARS Crop Improvement and Protection Research Unit, Salinas, CA 93905, USA; Department of Plant Pathology, University of Arkansas, Fayetteville, AR 72701, USA

## Abstract

Commercial production of spinach (*Spinacia oleracea* L.) is centered in California and Arizona in the US, where downy mildew caused by *Peronospora effusa* is the most destructive disease. Nineteen typical races of *P. effusa* have been reported to infect spinach, with 16 identified after 1990. The regular appearance of new pathogen races breaks the resistance gene introgressed in spinach. We attempted to map and delineate the *RPF2* locus at a finer resolution, identify linked single nucleotide polymorphism (SNP) markers, and report candidate downy mildew resistance (*R*) genes. Progeny populations segregating for *RPF2* locus derived from resistant differential cultivar Lazio were infected using race 5 of *P. effusa* and were used to study for genetic transmission and mapping analysis in this study. Association analysis performed with low coverage whole genome resequencing-generated SNP markers mapped the *RPF2* locus between 0.47 to 1.46 Mb of chromosome 3 with peak SNP (Chr3_1, 221, 009) showing a LOD value of 61.6 in the GLM model in TASSEL, which was within 1.08 Kb from Spo12821, a gene that encodes CC-NBS-LRR plant disease resistance protein. In addition, a combined analysis of progeny panels of Lazio and Whale segregating for *RPF2* and *RPF3* loci delineated the resistance section in chromosome 3 between 1.18–1.23 and 1.75–1.76 Mb. This study provides valuable information on the *RPF2* resistance region in the spinach cultivar Lazio compared to *RPF3* loci in the cultivar Whale. The *RPF2* and *RPF3* specific SNP markers, plus the resistant genes reported here, could add value to breeding efforts to develop downy mildew resistant cultivars in the future.

## Introduction

Spinach (*Spinacia oleracea* L.) is a leafy vegetable crop cultivated on 58, 000 acres in the US valued at more than 500 million dollars in farmgate value [1]. The US ranks second in spinach production after China. Spinach is primarily produced in California and Arizona in the US, favored by cool and dry environmental conditions. High nutrition content and other health-promoting compounds make spinach an excellent component of a healthy diet [2]. Demand for spinach is continually increasing in the US, particularly for organically produced spinach, which now accounts for nearly half of the total production.

Spinach, a diploid crop comprised of six chromosome pairs, is primarily a dioecious crop, although some other sex forms naturally occur. Selfing is not uncommon in female spinach plants, especially in the absence of pollen, which generates inbreed seeds lacking the desired combination of complementing *RPF* (Resistance to *Peronospora farinosa*) loci in hybrid seeds. Commercial seeds of spinach are produced in geographical regions with long days, moderate-cool temperatures, and dry weather during critical stages of pollination and seed set [3]. Optimal conditions for these stages are found in Denmark, Washington, and Oregon, where long days and minimal rainfall prevail. Denmark holds the largest share of spinach seed production, accounting for over 70% of global production, while Washington and Oregon contribute approximately 20%.

Downy mildew infection makes plants unsalable. Obligate oomycete *Peronospora effusa*, formerly *Peronospora farinosa,* causes downy mildew disease [2, 3] and is host-specific as it is only known to infect spinach. Nineteen distinct *P. effusa* races are currently known in spinach [4–6], with 16 of them documented after 1990. These new *P. effusa* races have been overcoming newly introgressed resistance genes in spinach cultivars. Thus, managing downy mildew disease is a major agenda of public and commercial breeders. Multiple *RPF* loci are hypothesized to provide resistance to different *P. effusa* races [7]. Most spinach hybrid cultivars contain complementing *RPF* loci from two inbred parents that offer resistance against many pathogen races. Despite the development of spinach cultivars with resistance to specific downy mildew races, no cultivar is known to be resistant to all races of the pathogen except for Sunangel, which is marketed as having resistance to races 1 through 19, although exhibiting intermediate resistance to race 10. Developing a spinach cultivar that can resist all available pathogen races would significantly benefit sustainable production until the appearance of new pathogen races. The search for new *RPF* genes that can resist new races of *P. effusa* remains a routine task of spinach breeders to minimize yield loss from downy mildew pathogens. A large number of germplasm comprising both cultivated and wild species are maintained in the National Plant Germplasm System of the United States Department of Agriculture (NPGS-USDA) and the Centre for Genetic Resources, the Netherlands (CGN), Wageningen University and Research (WUR) [3]. These accessions provide valuable genes for the improvement of spinach, including leaf quality, yield, and tolerance to abiotic and biotic stresses [3, 8, 9]. Strikingly, wild species of spinach have been the main source of disease-resistance genes, including resistance to downy mildew pathogens. Our lab has recently sequenced these cultivated and wild species maintained at the CGN-WUR, which will reveal new molecular tools for selecting resistance to multiple pathogens and other traits. Further, these new genomic resources may indicate the origin of current resistance (*R*) genes deployed in spinach cultivars that are kept confidential by commercial breeders.

Marker DM1 was established to locate 1.7 cM away from the *RPF1* locus in chromosome 3 [10]. Additional loci *RPF1*, *RPF2*, and *RPF3* were later mapped to a 1.5 Mb of spinach chromosome 3, and diagnostic markers to determine resistant-susceptible alleles were reported [11]. The *RPF1* locus was further narrowly mapped to 0.89 Mb of Sp75 chromosome 3 between 0.34–1.23 Mb [12]. Genotyping by sequencing (GBS) based SNP markers mapped the *RPF1* locus in a multi-parent progeny population to 0.84 Mb of Sp75 assembly [13]. Similarly, GBS markers mapped another loci resistance to *P. effusa* race 16 to a 0.57 Mb region of Sp75 assembly [14]. Downy mildew pathogen resistance from field evaluations in the diverse germplasm panel showed R genes between 0.3 to 1.5 Mb of chromosome 3 in Monoe-Viroflay genome assembly [15]. The *RPF3* locus in Whale was located at 1.25 Mb and at 2.73–2.74 Mb in Monoe-Viroflay genome assembly, and three regions of Sp75 genome assembly located at 1.19, 1.22–1.23, and 1.75–1.76 Mb [16]. The *RPF2* locus was recently reported to be between 1.11 to 1.72 Mb on chromosome 3 of Sp75 genome assembly [17].

Host genetic resistance continues to be the primary method for managing downy mildew disease in spinach, especially in organic production, despite the widespread use of chemical fungicides for managing various crop diseases. Identification of additional unique resistance materials effective against many races of downy mildew pathogens*,* plus deep molecular and genomic studies of genetic resistance mechanisms, may provide new tools to support precise *R* gene pyramiding. The downy mildew pathogen-resistant *RPF* genes have been breaking down with regularly emerging new races of *P. effusa.* This scenario has urged an in-depth characterization of the spinach-downy mildew pathogen interaction in disease development that may enable optimum use of host genetic resistance. Genome wide association studies (GWAS) are commonly used to identify chromosomal regions associated with trait expression in self-pollinated and cross-pollinated crop species. GWAS have been used to map the disease resistance regulating genomic regions and have identified single nucleotide polymorphism (SNP) markers that are associated with many traits in spinach [13, 15, 16, 18, 19]. In this study, we employed the low coverage whole genome resequencing method, also known as skim resequencing, to generate SNP genotype datasets. We selected this method as a continuation of our previous report [16] because of its capability to offer complete genome coverage, which is advantageous for genotyping compared to the GBS method commonly used in spinach genotyping studies [13, 14, 20], which is limited by incomplete genome coverage. Skim resequencing has been extensively applied in various crop species for discovering genome-wide genetic variants, particularly SNPs, and in trait mapping studies in diverse and bi-parental populations [21–24].

Spinach differential Lazio constitutes *RPF2* and *RPF4* loci that resist *P. effusa* races 1–10 and 15. Whale has an *RPF3* locus that provides resistance to races 1, 3, 5, 8–9, 11–12,14, 16, and 19, while Viroflay is entirely susceptible to all known races of downy mildew pathogen [4–6, 25]. Of the *RPF2* and *RPF4* loci in Lazio, only the *RPF2* locus resist race 5 of *P. effusa*, while the *RPF4* locus is susceptible; thus, the progenies of Lazio segregating for race 5 will uniquely map the *RPF2* locus in Lazio. Cross-bred progeny of Lazio and Viroflay screened for resistance to race 5 of *P. effusa* were used to map the *RPF2* locus. In addition, progenies of Viroflay x Lazio plus Viroflay x Whale populations were combined in order to map the overall resistance regulating region. This work identifies markers and genes associated with *RPF2* and *RPF3* loci and adds genomic resources to facilitate molecular-guided breeding in spinach.

## Results

### Resistance response in spinach progeny panel

In all inoculation experiments, the parental lines Lazio, NIL2, and NIL3 showed resistant reactions to *P. effusa* race 5 with no sporulation on cotyledon and true leaves, while the Viroflay and NIL4 were susceptible. The F2 seedling progenies from the cross of Viroflay x Lazio segregated upon inoculation, with 234 scored as resistant and 94 as susceptible. The segregation of Viroflay x Lazio progeny for *P. effusa* resistance in this experiment fit a 3:1 Mendelian segregation ratio (χ^2^ = 2.34, *P* = 0.13) which is expected for a trait regulated by a single dominant gene. For the Viroflay x Lazio panel, 192 seedlings (comprising 142 resistant and 48 susceptible seedlings and two parents) were sequenced to generate genotype datasets. In addition, 192 progeny panels segregating from Viroflay x Whale for race 5 of *P. effusa* reported earlier [16] were included to perform a meta-analysis.

### Variant discovery

Illumina sequencing of 192 Viroflay x Lazio progeny population generated 173.79 Gb sequence data. There were 1158.57 million raw reads, averaging 6.03 million reads per sample, equaling 1.01x average genome coverage. High-quality nucleotide bases with a Q score of 30 (>Q30) comprising 120.48 Gb data containing 810.06 million sequence reads were aligned against Sp75 reference genome assembly by implementing Illumina Dynamic Read Analysis for GENomics (DRAGEN) pipeline (v3.8.4), and for SNP calling. Raw SNP datasets were filtered for read depth to retain a minimum depth of coverage of 3 (DP 3), minimum genotype quality value of 9 (GQ 9), minor allele frequency (MAF) value of 0.05, and SNP with missing rates >75% using BCFtools [26] that retained filtered dataset comprising 617 998 SNPs with missing values of 68.01%. Beagle imputation retained 602 928 SNPs distributed in six chromosomes of spinach. This SNP dataset was filtered again to eliminate monomorphic SNPs, keep only biallelic variants, and exclude those with a missing rate > 25%, heterozygosity rate > 30%, and MAF value <5%. Further removal of non-polymorphic genotype calls in parents (identical calls in both Viroflay and Whale) retained 15 021 SNPs in six spinach chromosomes that were used for GWAS analysis.

Apart from Viroflay x Lazio progeny panel, SNPs were also discovered for the 384 progeny by merging another progeny panel of Viroflay x Whale reported previously [16], and we retained 34 234 SNPs for downstream GWAS analysis.

### Phylogenetic and population structure of progeny panel

Genetic diversity assessment revealed two main subpopulations in the Viroflay x Lazio progeny panel (Supplementary Figure 1). In contrast, the larger panel of the progeny of Viroflay x Lazio and Viroflay x Whale differentiated into four subpopulations, as observed in the PCA plot and NJ dendrogram produced by the GAPIT program (Supplementary Figure 2). Thus, two and four principal components (PC) were computed in both TASSEL and GAPIT programs and added as fixed effect factors during GWAS analysis to control for population structure.

### Fine mapping the *RPF* resistance region

**Figure 1:**
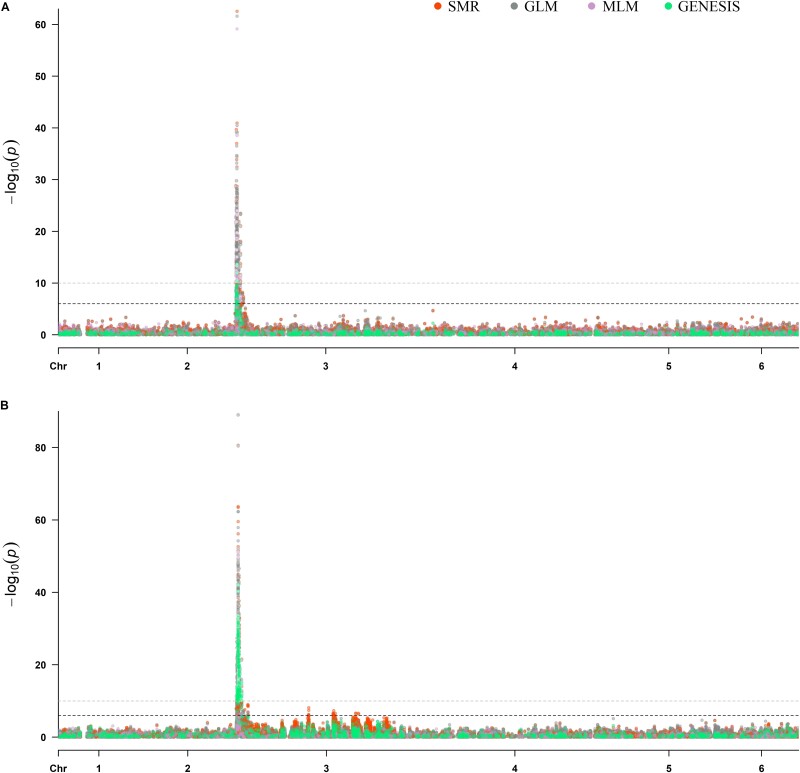
Manhattan plots of resistance to downy mildew pathogen (*P. effusa* race 5) in the progeny panel of Lazio x Viroflay segregating for the *RPF2* locus (A) and in combined progeny panel of Lazio x Viroflay and Whale x Viroflay segregating for the *RPF2* and *RPF3* loci (B). GWAS analysis in this study was performed with single marker regression (SMR), general linear model (GLM), mixed linear model (MLM) in the TASSEL program and logistic mixed model (LMM) in the GENESIS program. Markers associations from different models in the Manhattan plot are indicated with unique colors. The horizontal axis displays the physical position of the SNP. The vertical axis displays the association of the SNP with the trait expressed as -log_10_(*P*-value). The Sp75 assembly was used as the reference to call SNPs used in this study.

**Figure 2:**
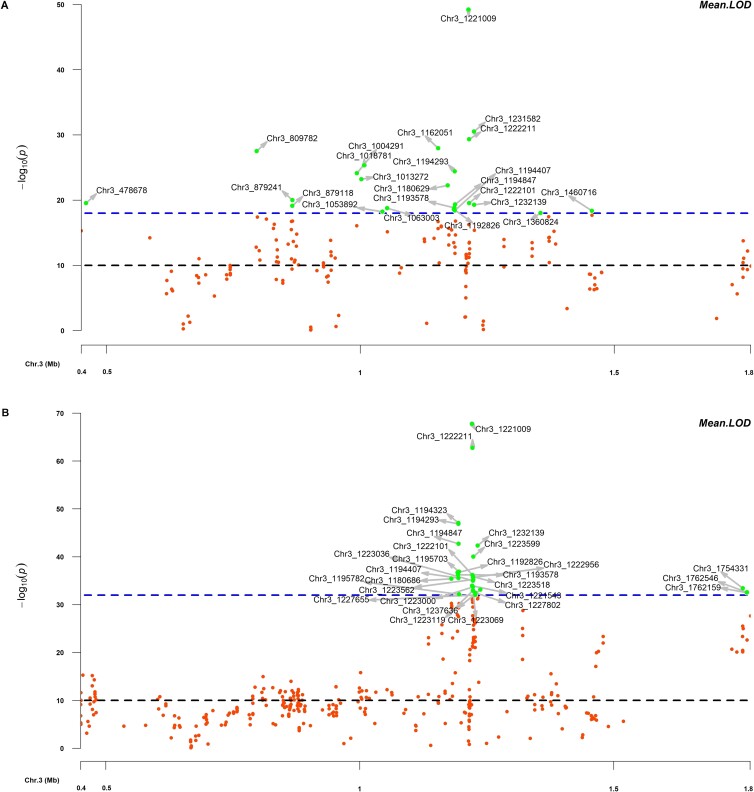
The *RPF* loci have been mapped between 0.40 to 1.80 Mb of chromosome 3 in Sp75. Association analysis maps *RPF2* locus in spinach progenies of Lazio x Viroflay segregating for race 5 of *P. effusa* (A). Regional association plot following the second GWAS analysis simultaneously mapped the *RPF2* and *RPF3* resistance region in a combined progeny panel of Lazio x Vrioflay and Whale x Viroflay (B). The horizontal axis represents the genomic position of the SNP and the vertical axis represents the strength of association of the SNP with the trait expressed as -log_10_(*P*-value). SNPs were identified based on the Sp75 assembly.

In the first set, association analysis was conducted among the 192 F2 seedling progenies of the Viroflay x Lazio population to map the *RPF2* locus. Twenty-three SNPs were identified to associate with the *RPF2* locus with a mean LOD value >18 across four GWAS models implemented in TASSEL and GENESIS programs (Table 1, Figure 1A), although more than 100 SNPs were observed with a LOD value >10. These 23 *RPF2*-associated SNPs were localized in chromosome 3 between 0.47 to 1.46 Mb. The strength of *RPF2*-associated SNPs was powerful as the peak-associated SNP (Chr3_1 221 009) showed the LOD values of 62.5, 61.6, 59.1 in TASSEL SMR, GLM, and MLM models and 13.6 in the GENESIS logistic mixed model. The mean LOD values of the 23 SNPs associated with the *RPF2* locus ranged from 18.02 to 49.21 and were located at 0.478, 0.809, 0.879, 1.00–1.06, 1.16–1.19, 1.22–1.23, 1.36, and 1.46 Mb of Sp75 chromosome 3 (Figure 2A). The proportion of phenotypic variance (R^2^ %) explained by the *RPF2-*associated SNPs in the SMR, GLM, MLM, and LMM GWAS models was 54.5–86.1, 54.4–85.8, 25.7–84.8, and 27.7–69.3 respectively (Table 1). The peak associated SNP Chr3_1 221 009 showed the highest R^2^ in all tested GWAS models.

**Table 1:** SNP markers associated with *RPF2* loci in Viroflay x Lazio progeny population inoculated with *P. effusa* race 5 in the first GWAS panel. In the second GWAS panel, the *RPF2* and *RPF3* resistance regions were analyzed by merging the two progeny panels Viroflay x Lazio and Viroflay x Whale; both inoculated with race 5

SNP^a^	Chr	Position	Allele^b^	MAF^c^	LOD (-log_10_*P*) value^d^					R^2^% value^e^				
					TASSEL			GENESIS	TASSEL				GENESIS	
					SMR	GLM	MLM	LMM	Mean LOD	SMR	GLM	MLM	LMM	Mean R^2^
*GWAS analysis in Viroflay x Lazio F2 progeny population*														
Chr3_478 678	3	478 678	**G**/T	0.43	28.83	28.60	12.05	8.66	19.53	59.97	59.83	32.11	42.68	48.65
Chr3_809 782	3	809 782	**T**/A	0.38	39.68	39.20	21.80	9.37	27.51	70.19	70.06	48.98	46.49	58.93
Chr3_879 118	3	879 118	**C**/A	0.38	28.32	28.38	11.44	8.36	19.13	58.33	58.63	30.11	41.12	47.05
Chr3_879 241	3	879 241	**A**/C	0.39	29.78	29.76	12.34	8.06	19.98	60.16	60.34	32.05	39.48	48.01
Chr3_1 004 291	3	1 004 291	**G**/T	0.34	34.63	34.63	18.41	8.78	24.11	60.65	60.81	39.42	43.33	51.05
Chr3_1 013 272	3	1 013 272	**A**/C	0.38	33.87	33.17	16.39	9.38	23.20	63.92	63.00	39.30	46.54	53.19
Chr3_1 018 781	3	1 018 781	**C**/G	0.39	36.97	36.47	18.67	9.17	25.32	67.61	67.58	43.81	45.46	56.12
Chr3_1 053 892	3	1 053 892	**G**/A	0.46	27.72	27.33	10.28	7.56	18.22	54.75	54.56	25.74	36.81	42.96
Chr3_1 063 003	3	1 063 003	**T**/C	0.48	27.66	27.19	11.77	8.45	18.77	56.04	55.68	29.81	41.61	45.78
Chr3_1 162 051	3	1 162 051	**T**/A	0.39	39.21	38.99	23.17	10.44	27.95	71.96	72.05	53.22	52.26	62.37
Chr3_1 180 629	3	1 180 629	**T**/G	0.46	32.43	32.11	15.03	9.48	22.26	62.09	61.76	36.57	47.08	51.87
**Chr3_1 192 826**	3	1 192 826	**C**/G	0.40	27.92	27.51	11.31	7.63	18.59	58.30	57.96	30.07	37.22	45.89
**Chr3_1 193 578**	3	1 193 578	**T**/C	0.40	28.07	28.23	10.84	8.09	18.81	58.01	58.49	28.65	39.65	46.20
**Chr3_1 194 293**	3	1 194 293	**C**/T	0.34	34.60	34.42	19.72	8.84	24.40	65.68	65.90	45.76	43.69	55.26
**Chr3_1 194 407**	3	1 194 407	**G**/C	0.39	26.83	26.59	12.14	8.07	18.41	54.48	54.44	30.27	39.53	44.68
**Chr3_1 194 847**	3	1 194 847	**T**/G	0.38	28.25	27.98	13.31	7.76	19.32	57.51	57.63	33.49	37.92	46.63
**Chr3_1 221 009**	3	1 221 009	**G**/A	0.41	62.52	61.59	59.12	13.61	49.21	86.08	85.80	84.83	69.34	81.51
**Chr3_1 222 101**	3	1 222 101	**G**/A	0.48	28.66	28.19	11.54	9.76	19.54	58.77	58.46	30.32	48.61	49.04
**Chr3_1 222 211**	3	1 222 211	**T**/C	0.43	40.92	40.45	23.98	11.96	29.33	72.50	72.51	53.54	60.45	64.75
Chr3_1 231 582	3	1 231 582	**G**/A	0.27	39.11	38.57	38.57	5.85	30.52	71.37	71.33	71.33	27.73	60.44
**Chr3_1 232 139**	3	1 232 139	**T**/C	0.45	27.95	27.86	11.84	9.48	19.28	55.72	55.94	29.46	47.08	47.05
Chr3_1 360 824	3	1 360 824	**G**/A	0.47	26.72	26.90	10.37	8.09	18.02	57.71	57.95	28.65	39.67	45.99
Chr3_1 460 716	3	1 460 716	**A**/G	0.41	27.27	27.49	11.33	7.35	18.36	58.70	59.33	30.96	35.69	46.17
*GWAS analysis in Viroflay x Lazio and Viroflay x Whale F2 progeny population*														
Chr3_1 180 686	3	1 180 686	**A**/C	0.47	48.13	47.49	17.83	28.18	35.41	51.08	49.09	23.53	44.98	42.17
**Chr3_1 192 826**	3	1 192 826	**C**/G	0.40	51.07	49.22	16.75	29.78	36.70	53.86	50.61	22.56	47.61	43.66
**Chr3_1 193 578**	3	1 193 578	**T**/C	0.40	49.06	48.55	17.95	29.45	36.25	51.75	49.71	23.59	47.07	43.03
**Chr3_1 194 293**	3	1 194 293	**C**/T	0.36	63.43	62.24	29.97	31.80	46.86	61.87	59.04	36.55	50.95	52.10
Chr3_1 194 323	3	1 194 323	**C**/T	0.37	63.66	62.30	29.97	32.23	47.04	62.12	59.05	36.76	51.66	52.40
**Chr3_1 194 407**	3	1 194 407	**G**/C	0.41	49.35	47.49	17.18	28.16	35.54	50.52	47.45	21.85	44.93	41.19
**Chr3_1 194 847**	3	1 194 847	**T**/G	0.39	59.52	57.86	22.01	31.51	42.73	58.23	55.76	27.78	50.48	48.06
Chr3_1 195 703	3	1 195 703	**C**/T	0.42	51.58	50.23	17.61	28.11	36.88	55.42	53.08	24.32	44.85	44.42
Chr3_1 195 782	3	1 195 782	**T**/C	0.42	44.86	43.59	14.55	25.59	32.15	50.01	47.84	20.34	40.70	39.72
**Chr3_1 221 009**	3	1 221 009	**G**/A	0.44	89.02	88.95	51.08	42.02	67.77	75.56	72.82	55.78	67.86	68.00
Chr3_1 221 543	3	1 221 543	A/**C**	0.49	44.02	44.50	17.48	29.76	33.94	49.24	48.02	23.73	47.59	42.14
**Chr3_1 222 101**	3	1 222 101	A/**G**	0.49	47.82	48.10	18.03	30.83	36.19	52.48	51.23	24.68	49.35	44.43
**Chr3_1 222 211**	3	1 222 211	**T**/C	0.45	80.60	80.37	49.64	40.27	62.72	71.58	69.71	54.25	64.95	65.12
Chr3_1 222 956	3	1 222 956	**G**/A	0.50	47.70	47.22	17.56	30.32	35.70	50.77	48.38	23.21	48.51	42.72
Chr3_1 223 000	3	1 223 000	T/**C**	0.49	44.67	44.40	16.66	28.99	33.68	47.63	45.62	21.67	46.32	40.31
Chr3_1 223 036	3	1 223 036	C/**T**	0.49	47.02	45.91	17.85	29.19	34.99	49.06	46.55	22.83	46.64	41.27
Chr3_1 223 069	3	1 223 069	A/**T**	0.48	44.57	43.26	15.43	28.27	32.88	47.24	44.59	20.07	45.13	39.26
Chr3_1 223 119	3	1 223 119	G/**A**	0.49	44.94	43.36	15.19	28.36	32.96	47.00	43.87	19.52	45.27	38.91
Chr3_1 223 518	3	1 223 518	**A**/T	0.49	47.96	47.01	16.83	31.54	35.84	52.46	49.50	23.20	50.53	43.92
Chr3_1 223 562	3	1 223 562	**G**/A	0.49	47.65	46.44	15.89	31.11	35.27	51.53	48.60	21.67	49.81	42.90
Chr3_1 223 599	3	1 223 599	**A**/G	0.48	52.58	52.01	22.57	32.97	40.03	55.03	52.82	29.25	52.89	47.50
Chr3_1 227 655	3	1 227 655	A/**T**	0.49	44.00	42.27	13.44	28.33	32.01	49.22	45.66	18.76	45.23	39.72
Chr3_1 227 802	3	1 227 802	A/**G**	0.48	43.91	41.75	15.28	28.92	32.46	50.69	46.90	21.97	46.19	41.44
**Chr3_1 232 139**	3	1 232 139	**T**/C	0.47	56.15	54.18	25.43	33.59	42.34	56.46	53.13	31.50	53.91	48.75
Chr3_1 237 636	3	1 237 636	A/**G**	0.48	44.49	43.29	16.31	28.42	33.13	49.72	47.23	22.37	45.38	41.17
Chr3_1 754 331	3	1 754 331	**G**/T	0.46	46.57	44.71	16.35	25.97	33.40	50.61	47.54	22.16	41.33	40.41
Chr3_1 762 159	3	1 762 159	**T**/C	0.43	44.02	42.91	17.78	25.27	32.49	48.00	45.72	23.47	40.18	39.34
Chr3_1 762 546	3	1 762 546	**T**/A	0.43	44.59	42.34	17.55	25.87	32.59	50.15	46.70	24.23	41.16	40.56

a SNPs are named based on their physical location (base pair coordinate) on Sp75 chromosome assembly. SNPs highlighted in bold were significantly associated in both association panels analyzed here.

b Beneficial alleles that contribute to disease resistance are highlighted in bold.

c MAF is minor allele frequency.

d LOD (-log_10_*P*) value from the SMR, GLM, and MLM models in TASSEL and logistic mixed model (LMM) in GENESIS.

e Percentage of phenotypic variation (R^2^ %) explained by SNPs generated by GWAS models implemented in TASSEL and GENESIS program.

The second set of GWAS performed in a larger panel of 384 progeny lines of Viroflay x Lazio and Viroflay x Whale evaluated against *P. effusa* race 5 identified 28 SNPs that were associated with a mean LOD value above 32 in four TASSEL and GENESIS models (Table 1, Figure 1B). The strength of association of the *RPF2* and *RPF3* resistance region from joint analysis of 384 lines was exceptional with LOD values in the range of 43.9–89.0, 41.7–89.0, 13.4–51.1, 25.3–42.0 for SMR, GLM, MLM model of TASSEL and GENESIS LMM model. The mean LOD values for the four tested GWAS models were in the range of 32.0 to 67.8. Of these *RPF*-associated SNPs, Chr3_1 194 293, Chr3_1 194 323, Chr3_1 194 847, Chr3_1 221 009, Chr3_1 222 211, Chr3_1 223 599, and Chr3_1 232 139 were associated with mean LOD values >40, while Chr3_1 221 009 and Chr3_1 222 211 were associated with LOD value of 67.8 and 62.8. All these 28 *RPF2* + *RPF3* associated SNPs were located on the proximal end of chromosome 3 in two regions between 1.18–1.23 and 1.75–1.76 Mb (Figure 2B). These 28 SNPs explained the phenotypic variance (R^2^ %) of 47.0–75.6, 43.9–72.8, 18.8–55.8, and 40.2–67.9 in the SMR, GLM, MLM, and LMM GWAS models. Two peak-associated SNPs, Chr3_1 221 009 and Chr3_1 222 211, explained 75.6 and 71.6% of the total phenotypic variance in the SMR model and averaged 68.0 and 65.1 across four tested models (Table 1).

**Figure 3:**
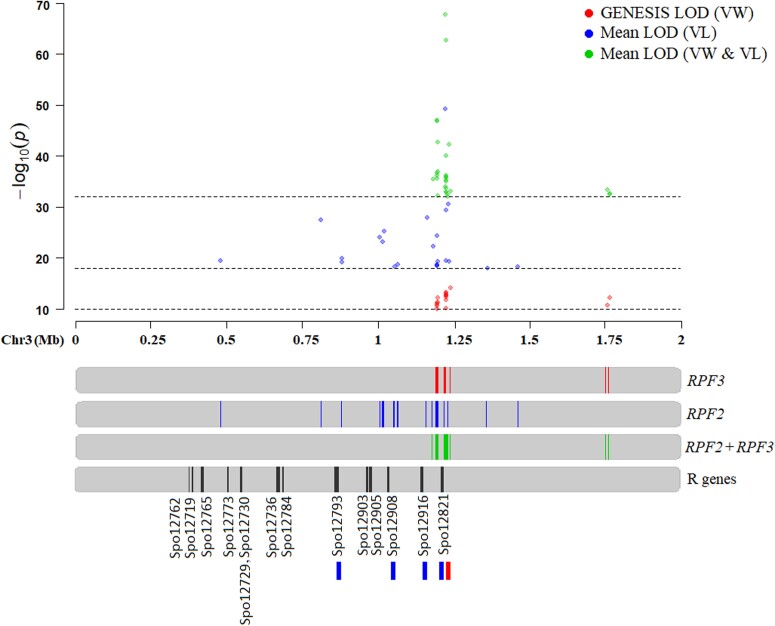
Overlay of the *RPF2* and *RPF3* associated region in spinach chromosome 3 and the disease resistance candidate genes. The physical location is based on the Sp75 assembly [27]. The red-filled circles in the Manhattan plot are the *RPF3*-associated SNPs based on the LMM model in the GENESIS program from the Viroflay x Whale population [16]. The blue-filled circles in the Manhattan plot are the *RPF2*-associated SNPs based on the mean LOD values across all four tested GWAS models from the Viroflay x Lazio progeny population in this study. The green-filled circles in the Manhattan plot are *RPF2* and *RPF3-*associated SNPs based on the mean LOD values across all four tested GWAS models in the combined progeny panel (Viroflay x Lazio and Viroflay x Whale) reported in this study. All known disease-resistance *R* genes in the overlaid region are presented as a black line. Resistant genes near the *RPF2* locus are marked with a blue line, while a red line denotes the gene in the vicinity of the *RPF3* locus. The R gene Spo12821 appears close to SNPs associated with *RPF2* and *RPF3* loci. SNPs near the Spo12793, Spo12908, and Spo12916 were only associated with the *RPF2* locus.

Combined GWAS analyses of 384 progenies of Viroflay x Lazio and Viroflay x Whale segregating for *RPF2* and *RPF3* loci were compared with individual panels to identify unique resistance regions for *RPF2* in Lazio and *RPF3* in Whale (Figure 3). Our previous study mapped the *RPF3* locus in cv. Whale within 1.19–1.23 and 1.75–1.76 Mb of Sp75 chromosome 3 [16]. The *RPF2* locus segregating from Viroflay x Lazio progenies did not show a strong association at the 1.75–1.76 Mb of chromosome 3; however, they were associated between 0.47 through 1.46 Mb (Figure 2A). On the other hand, a combined analysis of lines comprising both *RPF2* and *RPF3* loci showed an association in the 1.18–1.23 and 1.75–1.76 Mb regions (Figure 2B). Nine SNPs within 1.09 to 1.23 Mb of chromosome 3 (Chr3_1 192 826, Chr3_1 193 578, Chr3_1 194 293, Chr3_1 194 407, Chr3_1 194 847, Chr3_1 221 009, Chr3_1 222 101, Chr3_1 222 211, and Chr3_1 232 139) showed significant associations in the two association panel (Table 1). Overall, these result indicates that both *RPF2* and *RPF3* loci fall within 1.19–1.23 Mb of chromosome 3 (Figure 3). However, the *RPF2* locus was localized in a more extended region between 0.47–1.06 Mb and a common *RPF*-associated region of 1.19–1.23 Mb (Figure 3).

### Candidate genes associated with the *RPF* loci

Searching for resistance (*R*) genes in the proximity of the 23 *RPF2*-associated SNPs identified in the Viroflay x Lazio progeny revealed three genes within 10 Kb of the associated SNPs, plus Spo12908, located at 14–16 Kb away from the peak SNPs (Table 2, Figure 3). Gene Spo12793 encoding Serine/threonine-protein kinase was located at a six Kb distance from the *RPF2*-associated SNPs (Chr3_879 118, Chr3_879 241) in Sp75 assembly. Gene Spo12916 that encodes Leucine-rich repeat (LRR) was 8 Kb from the SNP marker Chr3_1 162 051. Gene Spo12821, a CC-NBS-LRR gene annotated to encode disease resistance protein, was within 1–2 Kb of SNPs Chr3_1 221 009, Chr3_1 222 101, and Chr3_1 222 211. The *RPF3*-associated SNPs between 1.22–1.23 Mb of chromosome 3 were at a distance of 2.41–2.65 Kb of the NBS-LRR gene Spo12821 [16], such that genes Spo12793, Spo12908, and Spo12916 lying between 0.87–1.16 Mb of Sp75 chromosome 3 appear to be more uniquely associated with the *RPF2* locus, as presented in Figure 3.

**Table 2:** Genes and their functions within 20 Kb of SNP markers that were identified to associate with the *RPF2* locus in spinach. SNPs highlighted in bold were significantly associated in both association panels analyzed here

*RPF2* associated SNPs			Gene info				Distance between SNP and gene (Kb)	
SNP	Chromosome	Position	Gene ID	Begin	End	Annotation	Begin	End
Chr3_478 678	3	478 678						
Chr3_809 782	3	809 782						
Chr3_879 118	3	879 118	Spo12793	861 496	873 154	Serine/threonine-protein kinase	17.622	5.964
Chr3_879 241	3	879 241	“	“	“	“	17.745	6.087
Chr3_1 004 291	3	1 004 291						
Chr3_1 013 272	3	1 013 272						
Chr3_1 018 781	3	1 018 781						
Chr3_1 053 892	3	1 053 892	Spo12908	1 037 102	1 039 775	Disease resistance protein	16.79	14.117
Chr3_1 063 003	3	1 063 003						
Chr3_1 162 051	3	1 162 051	Spo12916	1 147 645	1 154 005	Leucine-rich repeat (LRR)	14.406	8.046
Chr3_1 180 629	3	1 180 629						
**Chr3_1 192 826**	3	1 192 826						
**Chr3_1 193 578**	3	1 193 578						
**Chr3_1 194 293**	3	1 194 293						
**Chr3_1 194 407**	3	1 194 407						
**Chr3_1 194 847**	3	1 194 847						
**Chr3_1 221 009**	3	1 221 009	Spo12821	1 212 661	1 219 923	CC-NBS-LRR disease resistance protein	8.348	1.086
**Chr3_1 222 101**	3	1 222 101	“	“	“	“	9.44	2.178
**Chr3_1 222 211**	3	1 222 211	“	“	“	“	9.55	2.288
Chr3_1 231 582	3	1 231 582						
**Chr3_1 232 139**	3	1 232 139						
Chr3_1 360 824	3	1 360 824						
Chr3_1 460 716	3	1 460 716						

## Discussion

In this study, we aimed to map the *RPF2* locus in spinach, which is responsible for resistance against race 5 of downy mildew pathogen. Downy mildew is the most damaging disease of cultivated spinach in the US, Europe, and elsewhere. The downy mildew pathogen can cause severe damage to crops, rendering them unsalvageable. In particular, the problem is more severe when the crops are exposed to high humidity and overhead irrigation practices. This facilitates the pathogen’s spread across the entire acreage, resulting in massive crop losses. Breeding downy mildew pathogen resistant spinach varieties for commercial production is a continuous activity at public and private breeding programs by combining complementing *RPF* alleles from the two parents in the hybrid form [3, 7]. However, the effectiveness of *R* genes is limited for a pathogen that evolves rapidly; thus, the resistance effect is not durable. The regular emergence of new races and isolates of the downy mildew pathogen poses a challenge as it often overcomes the R genes deployed in spinach cultivars, making the new cultivars unable to provide long-term resistance against the pathogen. Thus, identifying the new *RPF* allele that is effective against recently emerged and predominant races of the downy mildew pathogen and developing molecular platforms to select resistant alleles are continuous activities in public and private spinach breeding programs. Continuous development of new markers to select each *RPF* locus is expected to enhance selection efficiency and expedite spinach breeding.

This study first mapped the *RPF2* locus segregating from breeding progenies of Lazio and Viroflay by employing multiple GWAS models to identify resistance locus-associated SNP markers as performed in our previous studies [13, 14, 16]. The GWAS analysis localized the *RPF2* resistance region within 0.47 to 1.46 Mb of Sp75 chromosome 3 based on the significance of association across tested GWAS models, including the LMM model in GENESIS. By reanalyzing the two progeny populations (Viroflay x Lazio and Viroflay x Whale) inoculated with the same pathogen race (*P. effusa* race 5), we mapped the *RPF2* and *RPF3* loci at fine resolution and differentiated the resistance-regulating region by individual *RPF2* and *RPF3* locus. The combined analyses showed associations for *RPF2* and *RPF3* loci at 1.18–1.23 Mb and 1.75–1.76 Mb. The *RPF3* locus was reported to localize on 1.19–1.23 and 1.75–1.76 Mb [16]. Thus, looking at the overlapping and unique genomic regions showing associations for the two *RPF* loci, we concluded that the *RPF2* locus extends through 0.47 to 1.06 Mb together with the 1.19–1.23 Mb region, the latter region associated even with the *RPF3* loci. The *RPF1* locus was mapped between 0.34–1.23 Mb [17] and 0.39, 0.69, 0.94–0.98, and 1.19–1.26 Mb of chromosome 3 based on Sp75 assembly [13]. The *RPF3* locus was assigned to three physical regions in 0.66–0.69, 1.05, and 1.22–1.23 Mb [14]. Association analysis of the germplasm panel evaluated in the field under natural infection by the downy mildew pathogen also found SNPs at 0.94, 1.06, and 1.16 Mb of chromosome 3 [18]. The three *RPF* loci (*RPF1*, *RPF2*, and *RPF3*) seem to be tightly linked, as all three loci were not stacked into one line by conventional crossing and have not been separated with the absence of recombination events in the region. The SNP markers reported here are helpful for marker assisted selection, especially in combination with other *RPF* genes. Further, incorporating *RPF* loci along with the field resistance QTLs [18] may increase the effectiveness of resistance genes. Additional investigations on developing markers flanking the *RPF* loci are underway to provide practical molecular tools for selection.

The spinach genome assembly shows clusters of *R* genes within the *RPF*-associated regions that accommodate six NBS-LRR genes between 0.6 and 1.3 Mb of Sp75 chromosome 3 [27], the same region with three *RPF* loci (*RPF1*, *RPF2*, and *RPF3*) are localized. This study mapped the *RPF2* locus in close proximity Spo12821 gene that encodes CC-NBS-LRR protein encoding, which was located at 1.08 Kb, 2.17 Kb, and 2.28 Kb of *RPF2*-associated SNPs: Chr3_1 221 009, Chr3_1 222 101, and Chr3_1 222 211. Similarly, other disease resistance genes were identified near the *RPF2* associated regions, such as Spo12793 encoding Serine/threonine-protein kinase was 5.96–5.08 Kb from SNP markers at 0.87 Mb, Spo12908 annotated as disease resistance protein was 14.11 Kb from SNP Chr3_1 053 892, and another gene Spo12916 with Leucine-rich repeat units was 8.04 Kb from SNP Chr3_1 162 051. Spo12821, an NBS-LRR encoding gene identified in the *RPF2*-associated region, was also reported as a potential candidate gene for *RPF3* locus [16] and *RPF2* locus [17], making this gene of interest to have a prominent role in providing resistance in spinach against *P. effusa*. The NBS-LRR is the most predominant class of *R* gene in plants that are known to act as a receptor of plant pathogen effector proteins and induces effector triggered immunity (ETI) by activating the downstream defense response to inhibit pathogen infection [28, 29]. Tandemly repeating LRR domains often recognize pathogen effector molecules and trigger resistance response [28, 30]. Recent reports have indicated structural variation in the length of the LRR regions of the Spo12821 gene between resistant and susceptible cultivars and the possible functional role of such variation in providing resistance response [17]. The disease resistance NBS-LRR genes are primarily found in clusters, as in the case of spinach chromosome 3. Multiple genes can complement in providing effective resistance, as in Arabidopsis against downy mildew pathogen *Hyaloperonospora arabidopsidis* (formerly *P. parasitica*) [31] and rice against rice blast pathogen *Magnaporthe grisea* [32]. The *RPF1-RPF6* have been established in spinach, plus molecular markers to select for *RPF1*-*RPF3* loci have been developed*.* However, the *R* genes have not been functionally validated. These genes reported in this study, most notably the Spo12821 consistent with both *RPF2* and *RPF3* loci, should be studied for structural and functional variation to elucidate the downy mildew disease resistance mechanism along with the involvement of other complementing genes in regulating resistance phenomena. New research aiming to characterize the functions of genes with gene editing and over-expression studies may explain the effective genes involved in resistance to downy mildew pathogens.

Low coverage genome sequencing (~1x) is a cost-effective approach to sequence a sample at a low coverage depth, typically between 1x and 3x, which can significantly reduce sequencing costs while still providing complete genome coverage and the ability to detect a greater amount of genetic variation. However, it is important to note that this approach is known to produce higher levels of missing data compared to higher coverage sequencing methods [24, 33]. To overcome this, high missing calls were imputed to determine the missing genotype calls or unobserved data in the samples using the haplotype profile. While genotype imputation can facilitate downstream applications by allele calling of poor quality and increasing marker density, lack of confidence in imputed genotype calls and high error rates remains a challenge. One limitation of low-coverage sequencing and imputation is that the heterozygote genotypes may be called as homozygous references. To address this, we filtered those imputed data by removing imputed calls with a genotype probability (GP) value below 90% (GP <0.90) to retain genotype calls imputed with high accuracy. However, we did not evaluate imputation accuracy and error level in this study. Further research on assessing the impact of imputation errors at different levels of sequencing depth (1x, 2x, 3x, and more) is necessary to determine the most promising practices for analyzing and utilizing low-coverage sequencing in heterozygous crops like spinach. However, we suggest employing coverage of 2-3x for effective allele calls.

Spinach cultivars containing multiple resistant genes may provide more durable resistance. Continued identification of new resistance sources, mapping new *RPF* genes, discovering markers, and gaining insights into their regulatory mechanism could present new and improved choices for breeding resistant cultivars. Such efforts are necessary to address the regularly emerging pathogen races that break the resistance loci. Overall, this study reports new sets of SNP markers to distinguish and select *RPF2* and the *RPF3* loci that can potentially accelerate the breeding process. The novel QTL regions and SNP markers identified here and the previously identified QTLs may have the potential to pyramid resistant genes when developing resistant spinach germplasm and breeding lines [10–14, 16–18]. In summary, the results of this study emphasize the significance of ongoing efforts in identifying and utilizing novel sources of resistance to develop spinach cultivars with improved resistance against downy mildew. The findings from this study provide new insights into the development of spinach cultivars with enhanced resistance to the downy mildew pathogen, ultimately benefitting both the growers and consumers of spinach.

## Conclusion

In conclusion, resistance to downy mildew pathogens is a key target trait in spinach breeding programs, but the frequent breakdown of resistance genes has been a major setback for spinach breeders. In this study, we successfully mapped the *RPF2* locus for resistance to downy mildew in the spinach cultivar Lazio within 0.47 to 1.46 Mb of chromosome 3, identifying 23 associated SNP markers with a mean LOD value >18 across four tested GWAS models. The peak associated SNP with the *RPF2* locus was located 2.41–3.65 Kb to the CC-NBS-LRR gene Spo12821, and other SNPs associated with the *RPF2* locus were found in proximity to genes Spo12793, Spo12908, and Spo12916, which are known to have disease resistance functions in plants. These *RPF2*-associated genes, especially Spo12821, offer promising prospects for future research to explore their role in regulating resistance against downy mildew pathogens and developing new tools and options for breeding resistant cultivars.

## Materials and methods

### Plant materials

The differential cultivars Lazio and Whale were crossed with Viroflay to generate F1 seeds. Lazio and Whale are resistant to race 5 of *P. effusa,* while Viroflay is susceptible to all races of *P. effusa*. The F1 male and female plants were allowed to inter-cross with F2 seeds harvested from female plants and used for inoculation and genetic analyses.

In the beginning, 10–20 F2 plants of Viroflay x Lazio plus parent lines were evaluated for disease reaction against race 5 of *P. effusa* (isolate UA201715) at the University of Arkansas. After initial screening and understanding of the segregation pattern from the small set of seedlings, the remaining F2 progenies of Viroflay x Lazio (n = 328) were inoculated. In all inoculation trials, parents, six near isogenic line (NIL) differentials (NIL1 through NIL6), and Viroflay were inoculated as controls along with the segregating progeny population. Seeds were sown in plastic trays (25 x 50 cm) filled with a potting mix (Sun Gro Horticulture, Canada). Around 10–15 seeds were sown in a greenhouse plant tray in ten rows. Seedlings were later thinned to 6–8 seedlings per row, and each seedling was labeled. Plants in trays were grown in the greenhouse (25°C) for two weeks.

In addition, 192 F2 seedlings of Viroflay x Whale segregating for resistance to *P. effusa* race 5 in our previous study [16] were merged with the Viroflay x Lazio population and reanalyzed here to map the *RPF* resistance region.

### Pathogen inoculation and disease screening

One leaf from every F2 seedling was excised before inoculation and stored for DNA extraction. All two-week-old seedlings in plant trays were spray inoculated using the previously reported whole-plant inoculation method [4, 5, 25]. Fresh inoculums prepared by washing off conidia from sporulated leaves of Viroflay plants were spray-inoculated on each plant, which was then incubated in a dew chamber (set at 18°C in the dark) for 24 h, a growth chamber (set at 18°C with 12 h dark–light cycle) for five days, and again in the dew chamber (set at 18°C) for 24 h. The disease reactions were observed for sporulation on cotyledons and true leaves and rated using a score of 0 to 4. A disease score of 0 = no sporulation; 1, 2, 3, 4 means up to 25%, 50%, 75%, and 100% leaf area with sporulation, as presented in the previous studies [16]. Each seedling was noted as “resistant” for a score of 0 or “susceptible” for a score of 1, 2, and 3 [16]. Disease responses were confirmed by re-inoculating and incubating for another week and re-scoring disease reaction after a week.

### Sequencing and variant calling

Sequencing and SNP variant calling were performed as previously described [16]. Genomic DNA was extracted using an automated KingFisher Flex extraction system (Thermo Fisher Scientific, Waltham, MA, USA), quantified using Qubit Fluorometer, sample integrity was determined based on 1% agarose gel electrophoresis, and sequenced at the genomics facility at Texas A&M. This study used the whole genome resequencing (WGR) method at a low coverage depth to obtain approximately 1 Gb of sequence reads per individual, expecting to provide 1x genome coverage sequences. Sequence reads were mapped to Sp75 reference assembly [27] and genotype calling by implementing Illumina Dynamic Read Analysis for GENomics (DRAGEN) pipeline v 3.8.4. Initially, SNPs were processed to filter low-quality calls by removing variants with a minimum coverage depth of 3 (DP 3), minimum genotype quality value less than 9 (GQ <9), minor allele frequency (MAF <0.05), and genotype calls with missing rate > 75% using BCFtools [26]. These filtered SNPs were then imputed by implementing the Beagle 4.1 [34] tool. Imputed datasets with genotype probability (GP) calls >0.9 were retained.

Next, the SNP dataset from six spinach chromosomes was extracted using BCFtools [26]. The genotype dataset was filtered to remove monomorphic loci, keep only biallelic loci, and remove indels and SNPs around ten bp of indels. The SNP dataset was further filtered to remove SNPs with missing calls >25% calls using BCFtools [26], heterozygosity rate > 30%, and allele frequency < 5%. Finally, SNP showing no polymorphism among two-parent lines, Lazio and Viroflay, were removed.

### Population structure and clustering

We swiftly assessed population structure and principal component analysis (PCA) using all SNPs in GAPIT3 [35, 36]. The PCA and unweighted neighbor-joining (NJ) tree were drawn in GAPIT for two sub-population for the Viroflay x Lazio progeny panel. Similarly, PCA and NJ trees for multi-parent progeny panels (comprising Viroflay x Laxio and Viroflay x Whale progeny) were drawn for four sub-population in GAPIT3. In addition, PCA analysis was internally performed in TASSEL [37] and GENESIS [38] programs to use as covariates in the GWAS analysis.

### Mapping the resistance region

GWAS analysis was initially performed in TASSEL 5.2.85 by implementing single marker regression (SMR), general linear model (GLM), and mixed linear model (MLM) [37]. The GLM and MLM models in TASSEL were run by including two PCA matrices and in-built kinship matrices. GWAS analysis was further performed by implementing logistic mixed model (LMM) that incorporates inbuilt PCAs and kinship matrices in the GENESIS R package [38]. The TASSEL models are more suitable to perform GWAS analysis of quantitative phenotype, while the LMM model in GENESIS is designed to fit the qualitative phenotype scores. Downy mildew disease score of 0 and 1 for a resistant and susceptible reaction was used in GWAS analysis in the GENESIS program, while disease response was converted to 1 for resistant and 9 for susceptible in TASSEL.

A meta-GWAS analysis was planned for a multi-parent population segregating for *P. effusa* race 5 by merging the Viroflay x Lazio population (described here) with the previous report of Viroflay x Whale population [16]. This meta-analysis aimed to simultaneously identify *RPF2* and *RPF3* associated regions following combined analysis and potentially identify unique R gene regions compared with the individual GWAS panels of Viroflay x Lazio (reported here) and the previous study comprising Viroflay x Whale progeny population [16]. For 384 progeny lines segregating from two populations, Viroflay x Lazio and Viroflay x Whale, SNPs were discovered following the above-outlined steps and were used for the GWAS analysis described above, including the first four principal components.

Manhattan plots and QQ plots were drawn in the R program using the CMplot package. A GWAS significance threshold of -log_10_(*P*-value) or the LOD score > 18 was employed to control false positives in Viroflay x Lazio progeny population. For the combined population of Viroflay x Lazio and Virolflay x Whale, a higher LOD score > 32 was set to consider the significance of the association.

### Candidate gene identification

For all *RPF*-associated SNPs identified across tested GWAS models, genes were searched around 20 Kb of associated SNPs in the Sp75 reference sequences [27]. Genes annotated to provide resistance in crops within 20 Kb of *RPF*-associated SNPs were considered candidate genes with likely involvement in providing resistance to the downy mildew pathogen. Predicted functions of such potential candidate genes in proximity (20 Kb) of *RPF*-associated SNPs were described.

## Supplementary Material

Web_Material_uhad076

## Data Availability

The genetic datasets generated in this study are publicly available in FigShare: https://doi.org/10.6084/m9.figshare.20441916.v2.
