# Supplementary material for: Skim resequencing finely maps the downy mildew resistance loci RPF2 and RPF3 in spinach cultivars whale and Lazio
Source: Hortic Res. 2023 Apr 19;10(6):uhad076. doi: 10.1093/hr/uhad076 (PMC10261881; doi:10.1093/hr/uhad076)
Supplement: Web_Material_uhad076 [file web_material_uhad076.zip › Supplementary captions.docx]

Supplementary information

Figure S1. Genetic structure of the spinach population segregating from a cross of spinach cultivars Lazio and Viroflay differentiated into two main sub-populations based on the principal component analysis (PCA) plot (A) and neighbor-joining (NJ) phylogenetic trees (B) in GAPIT3.

Figure S2. Genetic structure of the spinach population segregating from Lazio x Viroflay and Whale x Viroflay differentiated into four main sub-populations based on the principal component analysis (PCA) plot (A) and neighbor-joining (NJ) phylogenetic trees (B) in GAPIT3.
