# Supplementary figures and images for: Skim resequencing finely maps the downy mildew resistance loci RPF2 and RPF3 in spinach cultivars whale and Lazio
Source: Hortic Res. 2023 Apr 19;10(6):uhad076. doi: 10.1093/hr/uhad076 (PMC10261881; doi:10.1093/hr/uhad076)

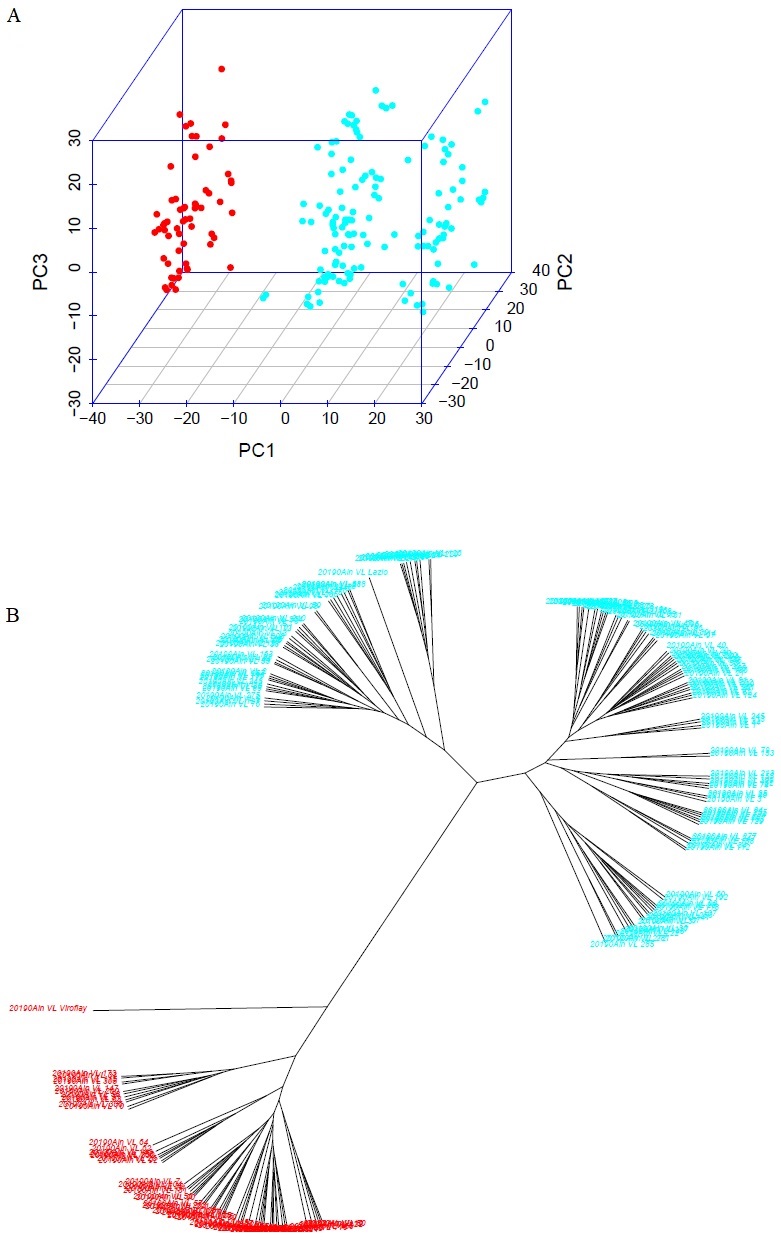

Supplement: Web_Material_uhad076 [file web_material_uhad076.zip › Figure S1.jpg]

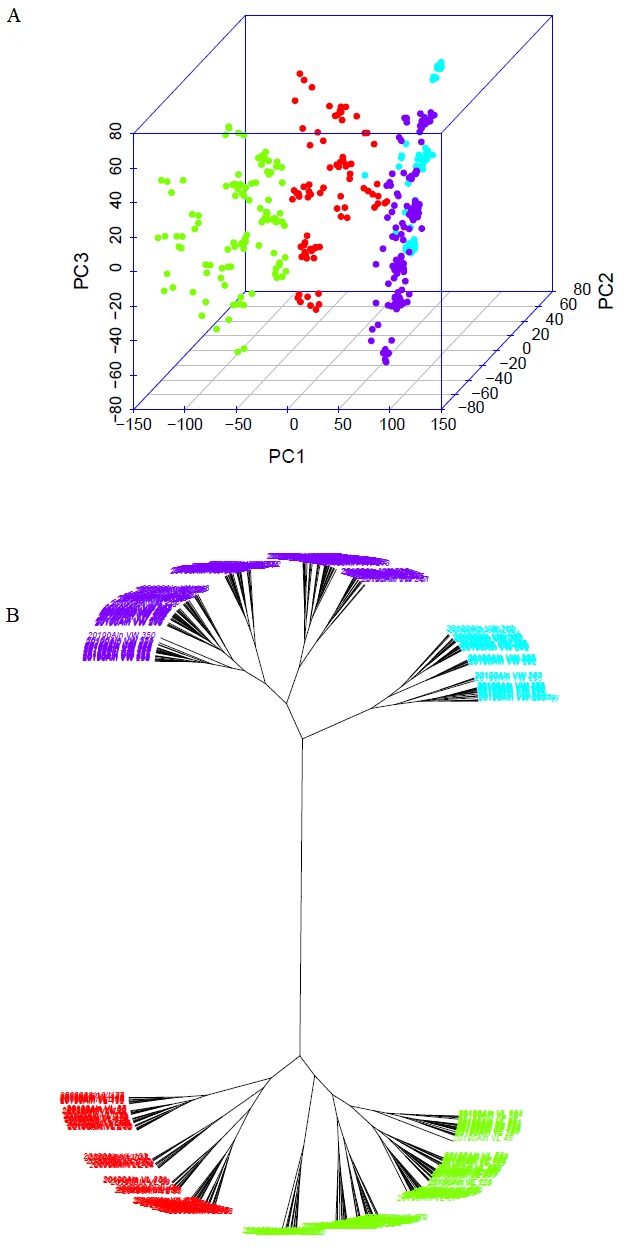

Supplement: Web_Material_uhad076 [file web_material_uhad076.zip › Figure S2.jpg]
